# Onychorrhexis and onychoschizia associated with ibrutinib therapy

**DOI:** 10.1016/j.jdcr.2025.09.039

**Published:** 2025-10-14

**Authors:** Angel Ray Baroz, Roberta Buist, Julie E. Mervak

**Affiliations:** a Pennsylvania State University College of Medicine, Hershey, Pennsylvania; b Department of Dermatology, University of Michigan – Michigan Medicine, Ann Arbor, Michigan

**Keywords:** brittle nails, Bruton tyrosine kinase inhibitor, BTK inhibitor, ibrutinib, nail dystrophy, onychorrhexis, onychoschizia, Waldenstrom macroglobulinemia

## Case description

**Fig 1:**
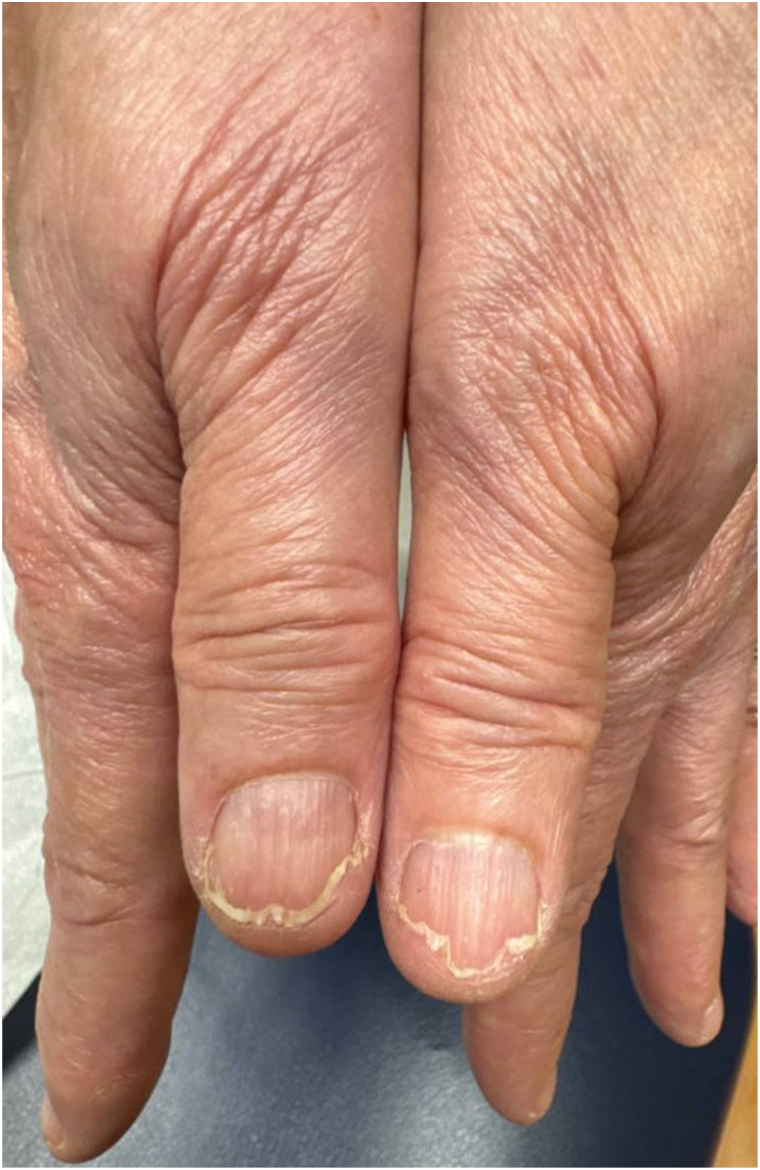
Longitudinal nail plate ridging consistent with onychorrhexis and distal lamellar nail plate splitting consistent with onychoschizia of the bilateral first fingernails.

**Fig 2:**
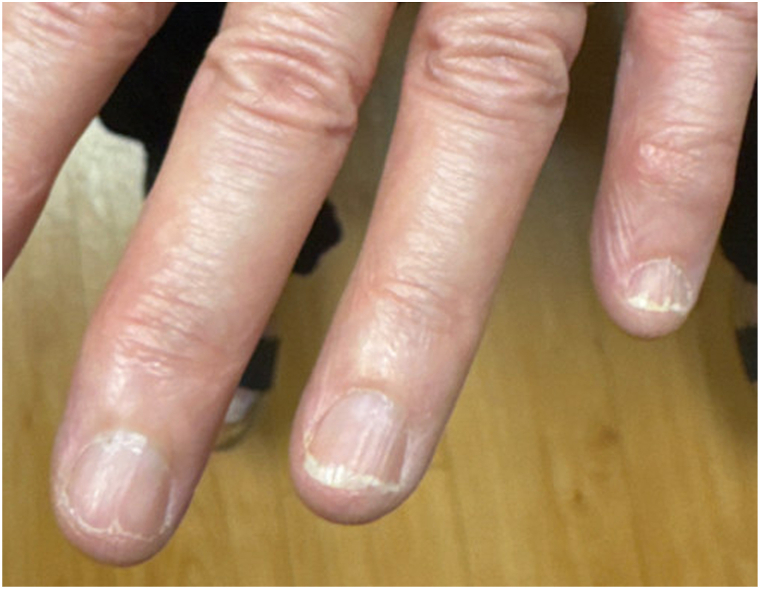
Longitudinal nail plate ridging consistent with onychorrhexis and distal lamellar nail plate splitting consistent with onychoschizia of the left third, fourth, and fifth fingernails.

A 75-year-old woman with Waldenstrom macroglobulinemia, diagnosed 6 years prior to our evaluation, presented to dermatology with progressive nail fragility (Figs 1 and 2). She had been receiving ibrutinib for 4 years with control of her hematologic disease. Nine months after initiating ibrutinib, she noted brittle nails characterized by distal nail plate splitting. She reported temporary improvement with daily application of strengthening nail lacquer and biotin supplementation.

Eight months prior to presentation, she contracted pulmonary coccidioidomycosis requiring long-term treatment with oral fluconazole. Following this, her nail brittleness worsened. She denied frequent water or other exposures. On physical examination, all 10 fingernails exhibited distal lamellar splitting, most pronounced in the bilateral first fingernails, consistent with onychoschizia, and longitudinal ridges, consistent with onychorrhexis. The toenails were less affected. There was no onycholysis, pitting, or subungual debris.

**Question: Which would be the most appropriate next step in management?**

**A.** Discontinue ibrutinib
**B.** Fungal culture
**C.** Hydrosoluble nail lacquer
**D.** Nail biopsy
**E.** Topical steroids

## Answer and discussion

Correct Answer: **C.** Hydrosoluble nail lacquer. Ibrutinib, a Bruton tyrosine kinase (BTK) inhibitor, is associated with nail toxicity, most commonly manifesting as brittle nails (onychoschizia and onychorrhexis). In a prospective study of patients with chronic lymphocytic leukemia on ibrutinib, brittle fingernails occurred in up to 67% of patients after a median of 6.5 months, and toenail changes occurred in 23% after a median of 9 months.^1^ These nail changes are believed to result from ibrutinib’s ability to covalently bind to cysteine 481, a cysteine residue found in both BTK and keratin.^2^ This disruption of disulfide bonds between cysteine residues impacts keratin integrity.^1^^,^^2^ The next-generation BTK inhibitors, acalabrutinib and zanubrutinib, although more specific to BTK inhibition with fewer off-target kinases impacted, appear to have a similar dermatologic toxicity profile.^2^

Brittle nails are a common complaint in older patients, particularly post-menopausal women. In this scenario the severity and timing point toward ibrutinib impacting the nail presentation, beyond normal aging alone. When counseling patients on treatment options, it is important for dermatologists to recognize that ibrutinib leads to a structural change and is not a consequence of inflammatory dermatoses. Nail lichen planus often presents with onychorrhexis and is included in the clinical differential; however, in this scenario, the nail changes are not secondary to lichenoid inflammation.

In this case, nail changes began 9 months after ibrutinib initiation, consistent with several published reports.^1^^,^^3^^,^^4^ Fluconazole is a potent inhibitor of cytochrome P450 3A (CYP3A), the primary metabolic pathway for ibrutinib. The patient’s worsening nail fragility after starting fluconazole raises the possibility of additive effects on nail keratinization in the setting of concurrent antifungal therapy. One pharmacokinetic study demonstrated a 1.2-fold increase in plasma ibrutinib concentrations during coadministration with fluconazole compared with ibrutinib alone.^5^

Management of ibrutinib-induced nail toxicity is symptomatic. Treatment options include cautious biotin supplementation, irritant avoidance, and topical nail-strengthening products such as hydrosoluble nail lacquers containing hydroxypropyl chitosan. Nail changes are typically reversible upon discontinuation of ibrutinib, although cessation is rarely pursued solely for this side effect.

## Conflicts of interest

None disclosed.
